# Spatiotemporal Dynamics of Inter-Item Competition in Visual Working Memory: The Role of Internal Attention

**DOI:** 10.3390/bs16101813

**Published:** 2026-10-04

**Authors:** Tengyi He, Taiwei Tan, Di Zhang

**Affiliations:** 1 Key Laboratory of Brain Function and Brain Disease Prevention and Treatment of Guizhou Province, Guiyang 550025, China; jiujiugu@gznu.edu.cn; 2 School of Psychology, Guizhou Normal University, Guiyang 550025, China; 3 School of Psychology, Capital Normal University, Beijing 100048, China; 2263501018@cnu.edu.cn

**Keywords:** internal attention, visual working memory, retro-cue, time course, spatial distance

## Abstract

Internal attention can increase the priority of task-relevant items in visual working memory; however, it remains unclear whether this effect is limited to improving target reports or also alters how inter-item influences evolve over time across different spatial distances. The present study employed a retro-cue color recall task to examine whether internal attention changes the distance-dependent temporal dynamics of inter-item influences in visual working memory. Valid retro-cues significantly reduced target report errors and shifted responses closer to the target relative to the non-target. Under neutral-cue conditions, inter-item influences exhibited distance-dependent temporal differentiation: the relative attraction index decreased significantly with increasing cue–probe interval (CPI) in D1 but increased significantly in D3, and the temporal slopes differed significantly between D1 and D3. In the valid-cue condition, none of the CPI slopes reached significance. However, direct cross-cue comparisons showed a significant difference in CPI slope only for D1, not for D2 or D3. These findings suggest that internal attention modulates the distance-dependent temporal pattern of inter-item influences as expressed in behavioral reports.

## 1. Introduction

Visual working memory (VWM) is a cognitive system that enables individuals to retain visual information for a short time and use it to guide subsequent behavior (Luck & Vogel, 2013; Van Ede & Nobre, 2023). VWM has a limited capacity (Adam et al., 2017; Cowan, 2001), and maintaining multiple representations requires allocating limited cognitive resources while prioritizing task-relevant items (Bays & Husain, 2008). Moreover, target and non-target representations interact and compete with each other (Oberauer & Lin, 2017; Wennberg & Serences, 2024). VWM performance therefore depends on both target accuracy and interactions among memory representations.

Internal attention, defined as the ability to direct attentional resources toward representations held in working memory, is a crucial mechanism for understanding the dynamic allocation of attention among multiple representations maintained in VWM (Chun et al., 2011; Kiyonaga & Egner, 2013). Internal attention can be retrospectively oriented toward internally represented locations or items after perceptual input is no longer available (Griffin & Nobre, 2003). Extensive research has shown that, during the retention interval, internal attention can be redirected to task-relevant items, thereby improving memory performance for cued items, an effect commonly referred to as the retro-cue benefit (RCB; Chen et al., 2025; Guo et al., 2026; Souza & Oberauer, 2016; Ye et al., 2020).

The retro-cue effects are not necessarily immediate but unfold over time following cue presentation (Guo et al., 2026; Pertzov et al., 2013; Schneider et al., 2016). In a study manipulating the stimulus onset asynchrony (SOA), the influence of non-cued items on subsequent processing was found to diminish gradually, with the N450 showing a marked attenuation approximately 400–600 ms after retro-cue onset (Schneider et al., 2016). Consistent with this temporal pattern, task-irrelevant features continued to produce attentional capture at short SOA, whereas this effect disappeared at longer SOA (G.-Q. Fu et al., 2026). At the neural level, retro-cues have also been shown to alter the temporal evolution of working memory representations, mitigating information loss during the retention interval (Ester et al., 2018). Therefore, examining final memory performance at only a single cue–probe interval (CPI) may obscure temporal changes in inter-item influences.

These changes in the influence of non-cued items raise a related question about the status of the underlying representations. The removal hypothesis posits that non-cued items are actively removed from VWM, whereas the prioritization hypothesis proposes that they remain at a lower priority than the cued item. Their status may also vary with cue reliability, task demands, and the stage of processing (Gunseli et al., 2015; Wennberg & Serences, 2024). Highly reliable cues may produce costs when non-cued items are subsequently tested (X. Fu et al., 2022). Nevertheless, non-cued items may still be retrieved or reused when they later become task-relevant (Gunseli et al., 2015; Van Moorselaar et al., 2015). Information rendered irrelevant by retrospective cueing can also exert attraction or repulsion effects on subsequent reports (Rhilinger et al., 2023). Neural evidence likewise suggests that information outside the focus of attention can remain represented, although its neural expression may vary. Latent visual working-memory representations can be restored later (Sprague et al., 2016), and information outside the current focus of attention can be transiently reactivated through perturbation when it remains potentially relevant (Rose et al., 2016). Complementing these reactivation findings, persistent activity in image-selective neurons of the human medial temporal lobe has also been reported for unattended working-memory items (Paluch et al., 2025). Thus, improved target performance does not necessarily imply that non-cued items have been removed from working memory.

Regardless of the neural state of non-cued items, as long as they remain partially activatable during retrieval or response selection, they may contaminate target reports. The Interference Model of Visual Working Memory posits that responses in continuous-report tasks are jointly determined by the target representation, other memory items, and noise (Oberauer & Lin, 2017). Memory items are typically bound to contextual information, such as spatial location. When the final test uses the target location as a retrieval cue, other items that share similar spatial contexts or are not clearly bound to their respective locations may also be partially activated. Consequently, uncertainty in identifying the appropriate memory item can increase swap errors, such that responses are based on non-target rather than target information (McMaster et al., 2022). Therefore, target-report errors may reflect not only imprecision in the target representation itself but also item confusion, binding uncertainty, and the relative weighting of different memory representations during retrieval and response output.

Spatial location plays a crucial organizational role in this process. Location information not only specifies where objects are located but also provides a contextual index for distinguishing, maintaining, and retrieving different memory items. Even when participants are not required to report locations, the original spatial positions of memory items may continue to organize internal selection. For example, Van Ede et al. (2019) found that selecting an item from VWM biased gaze toward the item’s memorized location, even though nothing remained there to look at. Similarly, De Vries et al. (2023) demonstrated that space continued to serve as an organizational scaffold for working memory even when temporal information could also be used to individuate memory items.

This spatial organization raises the possibility that proximity affects how readily items can be distinguished. Consistent with this possibility, studies of spatial transposition gradients have shown that locations near the target are more likely to be confused with the target during report (Emrich & Ferber, 2012; Rerko et al., 2014). In a more direct test of this idea, Liu et al. (2024) manipulated the spatial distance between cued and non-cued items and found that the influence of internal attention on non-cued items varied with distance: proximal and distal non-target items exhibited distinct patterns of memory cost.

Research on temporal processes and spatial distance collectively indicates that internal attention operates dynamically in time within a multi-item system organized by spatial relationships. Accordingly, the functioning of internal attention may follow distinct temporal patterns depending on the spatial arrangement of items. Spatial distance may determine not only the extent to which non-target items influence target reports at any given moment but also the manner in which this influence unfolds across the retention interval. However, it remains unclear whether inter-item influences at different spatial distances follow distinct temporal trajectories and whether internal attention alters these trajectories. Therefore, the study aimed to examine whether spatial distance governs the way inter-item influences evolve across the retention interval and whether establishing target priority through valid retro-cues reorganizes this distance-dependent temporal structure.

In summary, the present study proposed three hypotheses. First, valid retro-cues should reduce absolute report error and shift responses closer to the target relative to non-targets compared with neutral cues. Second, in the neutral-cue condition, inter-item influences should be jointly determined by spatial distance and CPI. Rather than forming a spatial gradient that remains constant throughout the retention interval, inter-item influences at different spatial distances may follow temporal trajectories. Finally, we hypothesized that valid retro-cues would alter the distance-dependent temporal trajectories observed in the neutral-cue condition. Together, these hypotheses addressed the primary objective of the present study: to examine how internal attention modulates the temporal dynamics of inter-item influences across different spatial distances.

## 2. Materials and Methods

### 2.1. Participants

The required sample size was approximated using G*Power (v3.1) (Faul et al., 2009) based on a repeated-measures analysis of variance. Drawing on the range of effect sizes reported in previous studies, the analysis assumed an effect size of *f* = 0.25, a significance level of *α* = 0.05, and a statistical power of 1 − *β* = 0.95. The analysis indicated that a minimum sample of 49 participants was required to detect the expected effect. A total of 53 adults were recruited through convenience sampling. All participants were right-handed, had normal or corrected-to-normal vision, and had normal color vision. Participants were also asked whether they had a history of psychiatric disorders or epilepsy and whether they had obtained sufficient sleep before the experiment; none reported psychiatric disorders or epilepsy, and all reported sufficient sleep. The same recruitment and screening criteria were applied throughout the study. One participant was excluded because their mean absolute report error (67.7°) was more than 3 *SD* above the group mean (*M* = 37.62°, *SD* = 8.13°; +3 *SD* cutoff = 62.02°), resulting in a final sample of 52 participants (20 men and 32 women; mean age = 20.5 years, *SD* = 2.29). All participants provided written informed consent before taking part in the experiment. The study was conducted in accordance with the Declaration of Helsinki and approved by the Committee for the Protection of Human Subjects at the School of Psychology, Guizhou Normal University (approval number: GZNUPSY.N.202510E [0016]).

### 2.2. Experimental Design

The study used a 2 (cue type: valid cue, neutral cue) × 6 (cue–probe interval, CPI: 0, 300, 600, 1000, 1800, 3000 ms) × 3 (target and non-target spatial distance: D1, D2, D3) within-subjects design. Hereafter, the interval between retro-cue offset and probe onset is referred to as the CPI, and the distance between the target and the relevant non-target is referred to as spatial distance.

**Figure 1:**
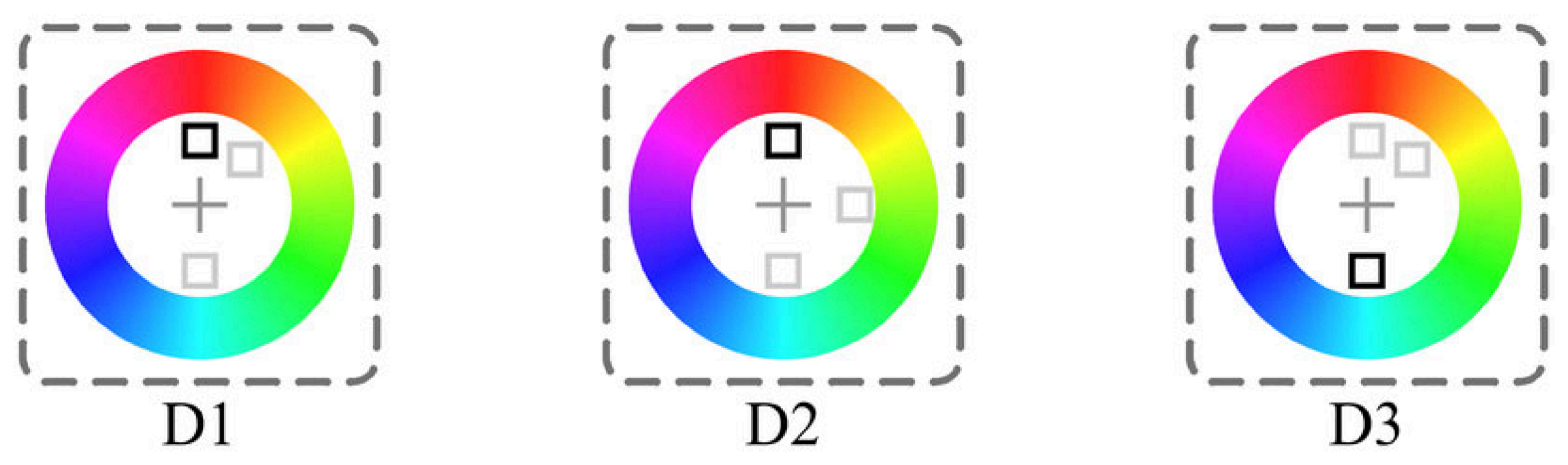
Illustration of the target and non-target spatial-distance conditions. The three memory items were presented at three locations selected from eight equally spaced positions around an imaginary circle. D1, D2, and D3 indicate that the target and its nearest non-target were separated by one, two, and three spatial steps, respectively. The configurations shown are for illustrative purposes only; the configurations varied across trials in the actual experiment.

In the valid retro-cue condition, the retro-cue indicated which item would subsequently be tested. In the neutral-cue condition, the cue provided no information about the identity of the target item. In the present study, the item ultimately tested was defined as the target item, whereas the remaining untested memory items were defined as non-target items. In the spatial-distance analyses, the relevant non-target specifically referred to the non-target item located closest to the target in space. The memory items were presented at eight equally spaced locations around an imaginary circle. D1, D2, and D3 indicated that the target and the relevant non-target were separated by one, two, or three spatial steps, respectively. The spatial configurations are illustrated in Figure 1.

All participants completed every experimental condition. Each participant completed a total of 360 experimental trials, which were presented in a randomized order. The primary dependent variables were absolute report error and the relative attraction index. Absolute report error quantified the overall accuracy of target-color reports, with lower values indicating greater accuracy. The relative attraction index quantified whether a response was relatively closer to the target or to a non-target. The index was calculated as d(T, R) − d(NT, R), where d(T, R) represents the circular distance between the target color and the reported color, and d(NT, R) represents the circular distance between the nearest non-target color and the reported color. Larger values indicate that the response was relatively closer to the non-target, whereas smaller or more negative values indicate that the response was relatively closer to the target.

### 2.3. Apparatus and Stimuli

Participants completed the experiment individually in separate testing rooms, with a fixed viewing distance of approximately 60 cm. Stimuli were presented against a white background (RGB: 255, 255, 255) on an LCD monitor with a resolution of 1920 × 1080 pixels, a refresh rate of 144 Hz, a display area of 60 × 33 cm, and a diagonal size of 69 cm. Stimulus presentation and response recording were controlled using PsychoPy (v2024.1.4).

The experimental stimuli consisted primarily of three differently colored square patches, each subtending approximately 2° × 2° of visual angle, and a response color wheel centered on the display, with a thickness of approximately 2.2° and a radius of approximately 8.2° of visual angle. The color wheel was defined in CIELAB color space and centered at L = 70, a = 20, and b = 38, following the general continuous-report approach used by Zhang and Luck (2008). The colors of the memory items were randomly selected from a set of 180 colors evenly distributed around the color wheel. The memory items were presented around an imaginary circle with a radius of approximately 4.5° of visual angle, and their locations were selected from eight equally spaced positions around the circle. On each trial, the response color wheel was presented at a random rotation to minimize the contribution of spatial memory to color reports.

### 2.4. Experimental Procedure

**Figure 2:**
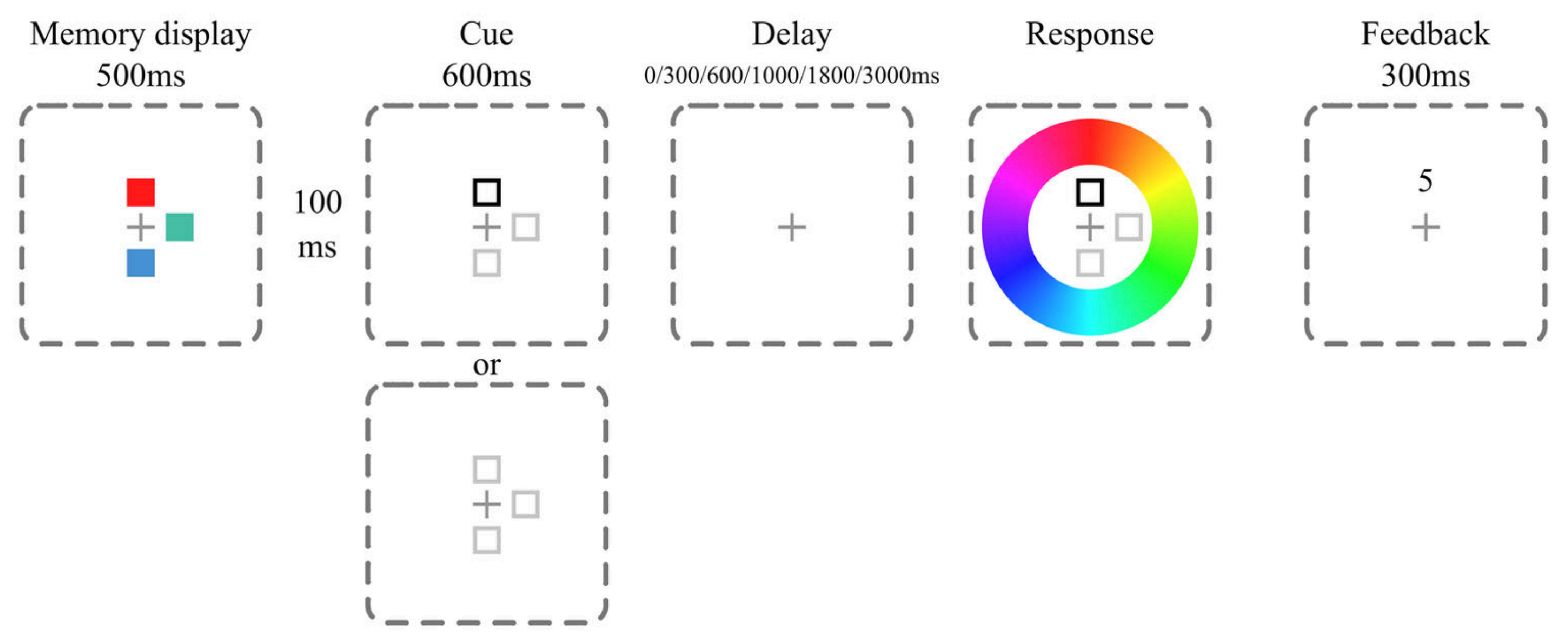
Illustration of a single experimental trial. Each trial began with a fixation cross presented for 100 ms, followed by a memory array containing three colored items for 500 ms. After a 100 ms blank interval, a retro-cue was presented for 600 ms. In the valid retro-cue condition, the target location was indicated by a thickened outline; in the neutral-cue condition, identical outlines were presented at all three locations and provided no information about which item would subsequently be tested. Following retro-cue offset, the continuous color wheel was presented after a CPI of 0, 300, 600, 1000, 1800, or 3000 ms. Participants reported the remembered color of the item at the probed location. The colors and spatial configuration shown are for illustrative purposes only.

The experimental procedure is illustrated in Figure 2. Each trial began with a fixation cross presented at the center of the screen for 100 ms, followed by a memory array consisting of three differently colored squares for 500 ms. After the memory array disappeared, a blank screen was presented for 100 ms, followed by a 600 ms retro-cue phase. In the valid retro-cue condition, the target location was indicated by a thickened outline; in the neutral-cue condition, identical outlines were presented at all three locations. A fixation cross remained on the screen during the post-cue delay, after which the response display appeared. Participants used the keyboard to select a color on the color wheel that matched their memory of the target color and then pressed the space key to confirm their response. After the response was submitted, feedback was presented for 300 ms, indicating the discrepancy between the reported color and the actual target color.

### 2.5. Data Analysis

In the present study, data were analyzed at the trial level using linear mixed-effects models. This approach is appropriate for repeated-measures or clustered data and is generally robust to violations of distributional assumptions (Baayen et al., 2008; Schielzeth et al., 2020). For absolute report error, cue type, CPI, spatial distance, and all interactions among these factors were included as fixed effects, with participant included as a random intercept. CPI was treated as a categorical variable to allow comparisons among the specific time points. For the relative attraction index, CPI was transformed as log(CPI + 1) and subsequently grand-mean-centered before being entered as a continuous predictor (DiMarco et al., 2023). This treatment was used to examine the overall temporal trend in the relative attraction index and differences in CPI slopes across experimental conditions. Cue type, spatial distance, CPI, and all interactions among these predictors were included as fixed effects, with participant included as a random intercept. To verify that the relative attraction index was not confounded by systematic differences in target–non-target feature separation, d(T,NT) was additionally analyzed as a function of cue type, CPI, and spatial distance, with participant included as a random intercept. An expanded model with by-participant random intercepts and random slopes for cue type, CPI, and spatial distance was fitted. This model yielded a singular fit and a convergence warning. The reported analyses retained by-participant random intercepts without random slopes.

## 3. Results

### 3.1. Absolute Report Error

**Figure 3:**
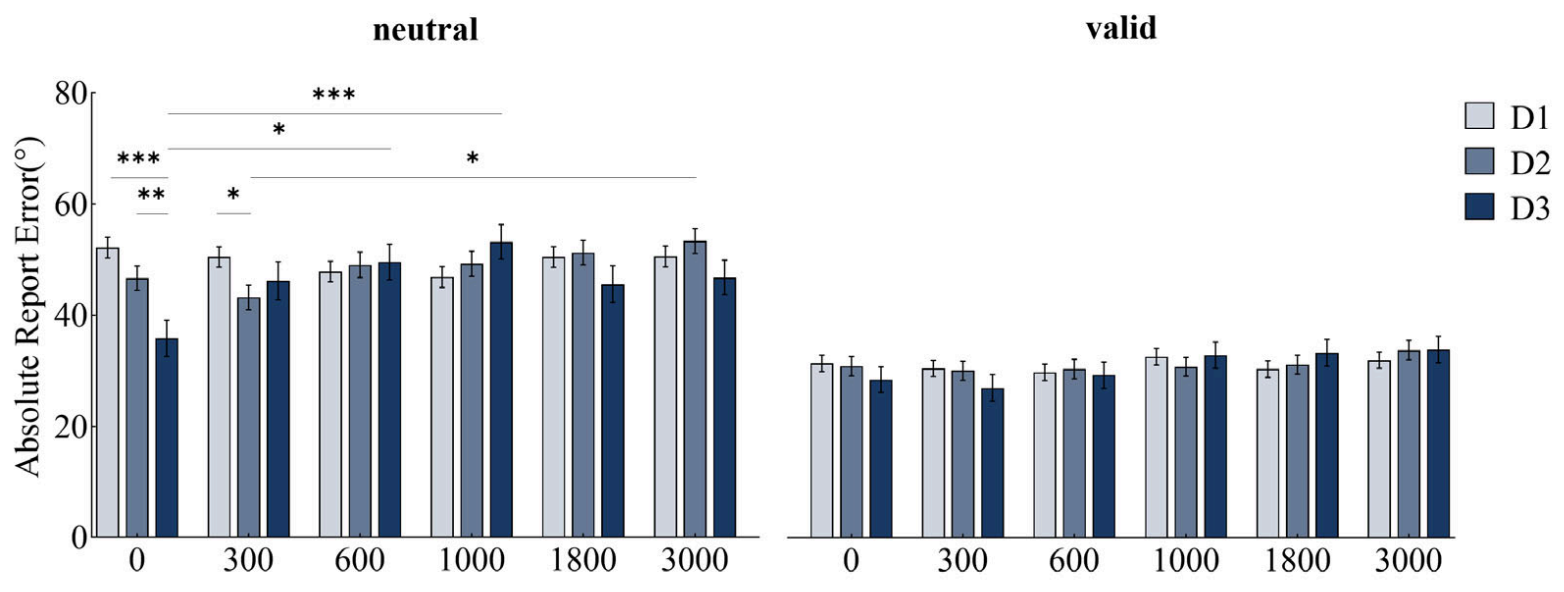
Absolute report error across cue types, spatial distances, and cue–probe intervals. The vertical axis represents the estimated marginal means of absolute report error derived from the linear mixed-effects model, and the error bars represent standard errors. The *p* values for post hoc pairwise comparisons were adjusted using Tukey’s method. * *p* < 0.05; ** *p* < 0.01; *** *p* < 0.001.

Absolute report error was analyzed using a linear mixed-effects model, with cue type, CPI, spatial distance, and all interactions among these factors included as fixed effects, and participants included as a random intercept (Figure 3). The main effect of cue type was significant: *F*(1, 18,635) = 656.37, *p* < 0.001. The main effect of CPI was significant: *F*(5, 18,635) = 4.12, *p* < 0.001. The main effect of spatial distance was not significant: *F*(2, 18,640) = 2.41, *p* = 0.090. In addition, the interaction between CPI and spatial distance was significant: *F*(10, 18,641) = 2.99, *p* < 0.001; the three-way interaction between cue type, spatial distance, and CPI was significant: *F*(10, 18,641) = 2.15, *p* = 0.018. The interaction between cue type and CPI, *F*(5, 18,635) = 0.79, *p* = 0.558, and the interaction between cue type and spatial distance, *F*(2, 18,641) = 1.75, *p* = 0.174, were not significant.

Estimated marginal means showed that absolute report error was significantly greater in the neutral-cue condition (*M* = 48.3, *SE* = 1.08) than in the valid retro-cue condition (*M* = 31.0, *SE* = 1.01), *p* < 0.001. Thus, valid retro-cue substantially improved the accuracy of target-color report.

Post hoc comparisons revealed that absolute report error was significantly lower in the valid retro-cue condition than in the neutral-cue condition across all combinations of spatial distance and CPI, except for the D3 condition at 0 ms, *p* = 0.054; all remaining *ps* < 0.05. In the neutral-cue condition, the effect of spatial distance was primarily observed at a CPI of 0 ms. Absolute report error was significantly lower in D3 than in D1, *p* < 0.001, and D2, *p* = 0.009. At a CPI of 300 ms, absolute report error was significantly lower in D2 than in D1, *p* = 0.014.

Further comparisons among CPI values within each spatial-distance condition revealed that, in the neutral-cue D3 condition, absolute report error was significantly lower at 0 ms than at 600 ms, *p* = 0.019, and 1000 ms, *p* < 0.001. In the D2 condition, absolute report error was significantly lower at 300 ms than at 3000 ms, *p* = 0.05.

### 3.2. Relative Attraction Index

**Figure 4:**
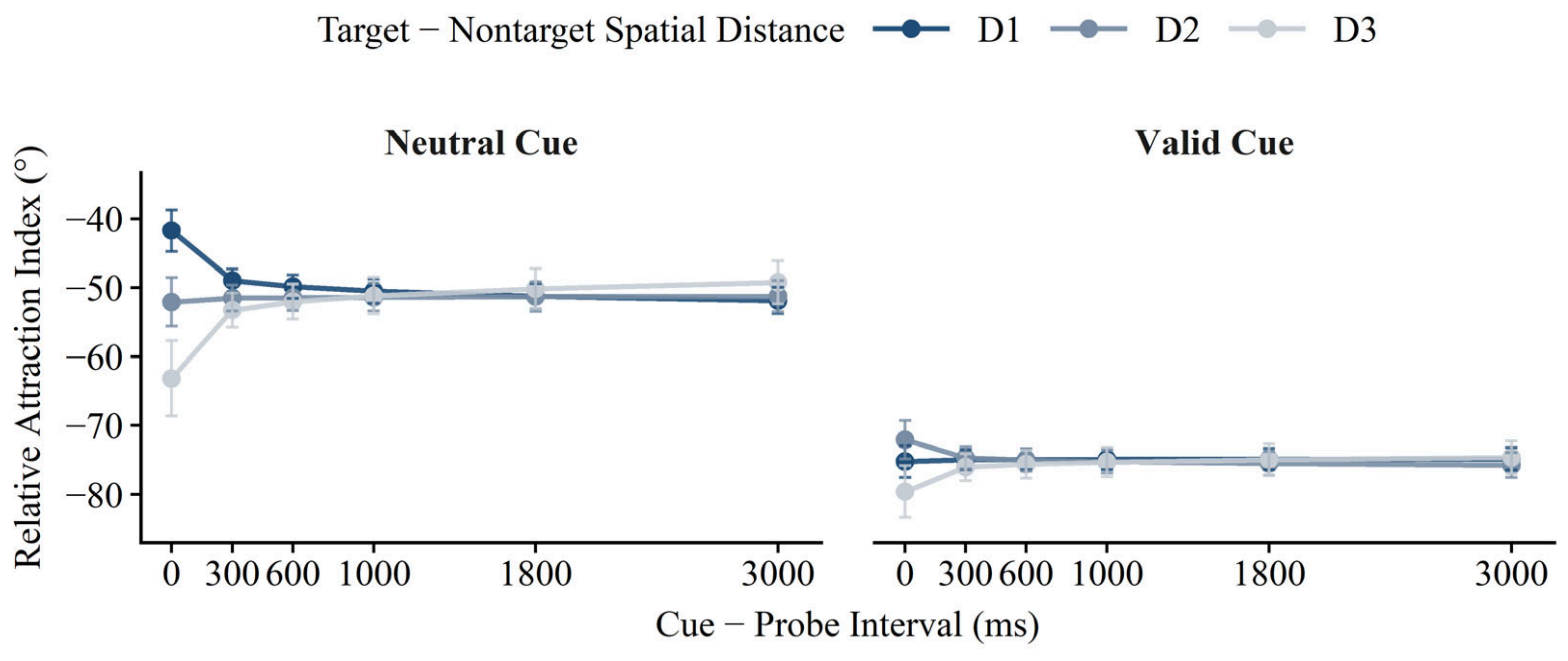
Relative attraction index across cue types, spatial distances, and cue–probe intervals. The left and right panels show the neutral-cue and valid retro-cue conditions, respectively. Data points represent the estimated marginal means derived from the linear mixed-effects model at each CPI, error bars represent standard errors, and connecting lines illustrate changes in the relative attraction index across CPI. The vertical axis shows the relative attraction index, and the horizontal axis displays the original CPI values.

A control analysis of target–non-target color distance, d(T,NT), revealed no significant main effects of cue type, CPI, or spatial distance, and none of the interactions among these factors reached significance, with all *ps* > 0.26. The relative attraction index was then analyzed using a linear mixed-effects model. Log-transformed, grand-mean-centered CPI was entered as a continuous predictor, cue type and spatial distance were entered as categorical predictors, and participant was included as a random intercept (Figure 4).

The main effect of cue type was significant: *F*(1, 18,661) = 412.60, *p* < 0.001. The interaction between spatial distance and CPI was also significant: *F*(2, 18,670) = 4.91, *p* = 0.007. Importantly, the three-way interaction among cue type, spatial distance, and CPI was significant: *F*(2, 18,670) = 3.63, *p* = 0.027; the distance-dependent pattern of temporal change in the relative attraction index differed between cue conditions. No other main effects or interactions reached significance.

Simple-slope analyses showed that, in the neutral-cue condition, the CPI slope was significantly negative for D1: *b* = −1.27, *SE* = 0.44, 95% CI [−2.13, −0.41]. Thus, as CPI increased, responses became relatively farther from the non-target and closer to the target. In contrast, the CPI slope was significantly positive for D3, *b* = 1.74, *SE* = 0.83, 95% CI [0.11, 3.37], indicating that responses became relatively closer to the non-target and farther from the target as CPI increased. The slope for D2 was not significant. The estimated difference between the D1 and D3 slopes was significant: estimated slope difference = −3.01, *SE* = 0.94, *p* = 0.004. The relative attraction index followed different temporal trajectories in D1 and D3 under neutral-cue conditions. In the valid-cue condition, none of the CPI slopes reached significance. Cross-cue comparisons further showed that the D1 slope differed significantly between the neutral and valid-cue conditions, with estimated slope difference = −1.31, *SE* = 0.53, and *p* = 0.014, whereas the corresponding differences for D2 and D3 were not significant.

## 4. Discussion

The present study used a retro-cue recall task to examine how internal attention modulates the spatiotemporal organization of inter-item influences in VWM. Valid retro-cues not only improved the accuracy of target reports but also shifted responses toward the target relative to non-target items, suggesting that internal attention can establish target priority. Under neutral-cue conditions, the relative attraction index exhibited distinct temporal trajectories across spatial distances over the retention interval, indicating that the relationships among multiple memory representations are dynamically organized in space and time. This distance-dependent temporal pattern differed between cue conditions.

Previous studies on retro-cues have shown that valid retro-cues can significantly improve target memory performance, producing the retro-cue benefit (Ester et al., 2018; Souza et al., 2016; Souza & Oberauer, 2016). In the present study, target prioritization was evident not only in reduced absolute report errors but also in a shift in responses toward the target relative to non-target items. A neural population-coding model indicates that the retro-cue benefit can be expressed as increased population activity associated with the cued item together with fewer exchange errors between the cued item and other items (Bays & Taylor, 2018). This model provides one possible account of the present behavioral pattern: the retro-cue benefit may involve both improved target-report accuracy and a reduced contribution of other items to the final report, resulting in a more pronounced target-centered response pattern.

Inter-item influences did not remain spatially fixed throughout the retention interval; instead, their temporal profiles differed across spatial distances. In the neutral-cue condition, the cue did not indicate which item would subsequently be tested, leaving all three items equally relevant during the post-cue retention interval. When memory was tested immediately after retro-cue offset, absolute report error was lower in D3 than in D1 and D2. One possible interpretation is that the spatially isolated item that was ultimately tested was more discriminable at retrieval during this early stage. The relative attraction index further showed that, in D1, early performance may have been disrupted by overlap in spatial context and uncertainty in feature–location binding, although this interference gradually diminished over time. In D3, by contrast, this early discriminability advantage may have weakened over time as responses gradually shifted toward the non-target. The contrasting temporal trajectories in D1 and D3 may jointly reflect changes in inter-item discriminability, the reliability of contextual binding, and the weights assigned during retrieval or response selection.

This interpretation is consistent with evidence for spatial transposition gradients, according to which items adjacent to the target location are more likely to be confused during reporting (Rerko et al., 2014). The binding between unattended representations and their locations also varies systematically with spatial distance, with greater spatial confusion at shorter cue–probe distances (Sahan et al., 2019). More broadly, the Interference Model of Visual Working Memory proposes that responses in continuous-report tasks are jointly determined by the target representation, non-target representations, and their contextual bindings rather than by the target representation alone. When the target location serves as a retrieval cue, overlapping spatial cues or uncertainty in contextual binding may partially activate other items and systematically bias target reports (Oberauer & Lin, 2017). Evidence that inter-item competition continues to constrain VWM during maintenance further supports this account (Wennberg & Serences, 2024). Uncertainty in contextual cues can also produce swap errors, in which a non-target is reported in place of the target (McMaster et al., 2022), and these errors are associated with identifiable neural representations (Mallett et al., 2022). Accordingly, non-target-related deviations in continuous-report tasks may reflect systematic competition or selection uncertainty among memory representations rather than a nonspecific increase in random error. The D1 cross-cue difference is also consistent with evidence that retro-cues can reduce the influence of non-cued information on subsequent processing (Schneider et al., 2016) and that retrospective attention can alter the temporal evolution of working-memory representations (Ester et al., 2018).

By contrast, differences in D2 and D3 CPI slopes between the valid retro-cue and neutral-cue conditions did not reach statistical significance. The present results therefore provide no reliable evidence that valid retro-cues reduced non-target-related bias in target reports. Thus, although retro-cues can establish target priority, information associated with non-targets may continue to influence the final report through mechanisms such as retrieval competition and spatial binding. Information rendered irrelevant by a retro-cue can continue to exert measurable attraction or repulsion effects on subsequent recall (Rhilinger et al., 2023). Improved target performance should therefore not be taken as evidence that all non-target representations have been completely removed.

The present findings do not fully align with the proximity-protection effect, according to which non-cued items adjacent to the cued target may retain greater accessibility (Liu et al., 2024). In the present study, by contrast, D1 items did not exert a stronger influence than more distant items in the valid retro-cue condition. One possible explanation for this discrepancy lies in differences in task demands and cue reliability. When non-cued items remain potential test targets, participants have reason to preserve their accessibility. In the present study, however, valid retro-cues consistently and accurately identified the final report target, making the other items irrelevant to the subsequent test. Participants can strategically adjust the maintenance status of non-cued items according to cue reliability, with highly reliable cues being more likely to be accompanied by reduced performance for non-cued items (Gunseli et al., 2015).

An additional difference concerns the dependent measure. The previous study examined whether non-cued items could still be reported directly, whereas the present study examined whether non-target items produced reliable and systematic biases in target reports (Liu et al., 2024). Residual accessibility does not necessarily mean that a non-target receives sufficient weight in the current response to produce a significant attraction effect. Evidence from visual-impulse perturbation and EEG decoding further suggests that cued and non-cued colors occupy different representational states following a retro-cue. The cued color was decodable from sustained alpha-band activity, whereas the non-cued color was not reliably decodable from sustained alpha-band activity; however, both colors could be decoded from neural activity evoked by visual impulses (Kandemir et al., 2024). These findings suggest that the non-cued color may no longer be maintained in the same sustained active state as the target but may remain in a lower-activity state that can be reactivated through perturbation (Kandemir et al., 2024). This account is consistent with evidence that latent representations can subsequently be reactivated (Rose et al., 2016; Sprague et al., 2016) and that prioritization can transform the representational format of working-memory contents (Pacheco-Estefan et al., 2024).

Taken together, one possible interpretation of the present behavioral findings is a flexible reorganization of the relative priority of memory representations following a valid retro-cue. However, the present behavioral measures cannot directly determine the neural representational states of non-target items. Moreover, removal and prioritization need not be mutually exclusive processing strategies across all task contexts. The functional state of non-target information may be jointly determined by cue reliability, task demands, spatial configuration, and the time elapsed since cue presentation (Gunseli et al., 2015; Liu et al., 2024).

Several limitations should be considered. First, the relative attraction index reflects the relative contributions of the target and non-target to behavioral reports but cannot independently determine whether the observed pattern reflects increased non-target influence, target degradation, or representational reweighting during maintenance, retrieval, or response selection. Second, the present study did not directly measure the accessibility or neural representational states of non-targets. Third, the present findings were obtained from a young convenience sample, and their generalizability to the broader adult population remains to be established. Fourth, omitting by-participant random slopes may affect fixed-effect inference if experimental effects vary across participants (Matuschek et al., 2017). Future research could draw on EEG decoding, eye-movement measures, perturbation-based decoding, and computational modeling to examine how non-target representations at different spatial distances change over the retention interval and to identify the cognitive mechanisms underlying these changes.

## 5. Conclusions

The present findings suggest that internal attention modulates the behavioral expression of inter-item influences over time in visual working memory, with the clearest direct evidence of cue-related modulation observed in D1. These findings extend the retro-cue literature by linking target prioritization to distance-dependent temporal patterns in memory reports.

## Data Availability

The data supporting the findings of this study are available from the corresponding author upon reasonable request.
